# Phenolic-Rich Extract from *Pyracantha fortuneana* Fruit Alleviates DSS-Induced Colitis in Mice: Insights from Gut Microbiota, Metabolomic, and Transcriptomic Analyses

**DOI:** 10.3390/foods15193528

**Published:** 2026-10-02

**Authors:** Hang Xu, Daiwei Xianyu, Wenwen Jin, Kezhuo Chen, Yinghua Deng, Jiaxin Li, Yuanmin Zhu, Chunhua Fu, Gongxuan Chen, Longjiang Yu

**Affiliations:** 1 Hubei Key Laboratory of Purification and Application of Plant Anticancer Active Ingredients, College of Chemistry and Life Sciences, Hubei University of Education, Wuhan 430205, China; 2 Institute of Resource Biology and Biotechnology, Department of Biotechnology, College of Life Science and Technology, Huazhong University of Science and Technology, Wuhan 430074, China; 3 Key Laboratory of Molecular Biophysics, Ministry of Education, Wuhan 430074, China

**Keywords:** *Pyracantha fortuneana* fruit, DSS-induced colitis, gut microbiota, multi-omics, transcriptome, bile acid metabolism

## Abstract

*Pyracantha fortuneana* (Maxim.) H.L.Li fruit is a bioactive dietary resource, yet its effects on intestinal inflammation and the associated microbial, metabolic, and host responses remain poorly understood. This study characterized *P. fortuneana* fruit extract (PFE) and evaluated its protective effects in a dextran sodium sulfate (DSS)-induced colitis model using microbiota, metabolomic, and transcriptomic analyses. Chemical profiling confirmed the presence of representative phenolic acids, flavonoids, and condensed tannins in PFE. Administered before and during 2.5% DSS exposure, PFE reduced disease activity, attenuated colon shortening and the elevated spleen index, and improved colonic histopathological appearance. PFE also reduced IL-6 and increased IL-10 and serum GSH-Px levels, while high-dose PFE decreased TNF-α. It restored ZO-1 expression, increased colonic diamine oxidase levels, and tended to increase Occludin expression. PFE increased microbial diversity and evenness, reduced *Turicibacter* and *Ruminococcus*, and increased *Prevotella* and *Jeotgalicoccus*. Non-targeted metabolomics identified changes in selected cecal bile acid- and lipid-related metabolites, whereas transcriptomic and protein analyses implicated bile secretion- and PPAR-related responses, accompanied by increased ASBT, PPARγ, and NLRP6 expression. Collectively, these findings demonstrate the protective effects of PFE against DSS-induced colitis and support its further investigation as a potential food-derived bioactive ingredient for intestinal health.

## 1. Introduction

Ulcerative colitis (UC) is a chronic inflammatory disease primarily affecting the rectum and colon, classified as a subtype of inflammatory bowel disease (IBD) [1]. In 2023, it is estimated that approximately 5 million individuals worldwide are afflicted by UC, with a rising incidence rate [1]. Common manifestations of UC include rectal bleeding, increased frequency of bowel movements, and altered stool consistency. When inflammation extends beyond the rectum, patients may experience tenesmus, fecal incontinence, and cramping abdominal pain, all of which significantly impact quality of life. Furthermore, prolonged UC increases the risk of colorectal cancer [2].

The pathogenesis of UC is not fully understood. Recent studies suggest that it may result from an exaggerated immune response to harmful exogenous agents, leading to increased inflammation, oxidative stress, disruption of the intestinal barrier, and dysbiosis of the gut microbiota. Key cytokines, particularly tumor necrosis factor (TNF) and interleukins (IL), are crucial in this process [3]. Oxidative stress, commonly observed in UC, arises from activated inflammatory pathways and the accumulation of reactive oxygen species [4]. Additionally, dysfunction of the intestinal barrier, characterized by reduced mucin-secreting goblet cells and tight junction proteins such as zonula occludens (ZOs) and occludin, exacerbates the condition [5,6]. Intestinal microbial communities contribute to host health, and disturbances in their composition are frequently observed in UC [7]. These disturbances can compromise the epithelial barrier and disrupt immune regulation [8]. Metabolites produced by intestinal microorganisms provide one route through which the microbiota influences host immune responses [9]. Dysbiosis alters metabolite profiles, impacting levels of bile acids, tryptophan, and short-chain fatty acids, which modulate immune responses through specific receptor activation [10]. Notably, secondary bile acids exhibit immunosuppressive properties [11]. Increased conjugated bile acids together with decreased secondary bile acids further highlight the link between microbiota composition and inflammatory status [12].

Current therapeutic strategies for UC, including aminosalicylates, immunosuppressants, and biological agents, are often limited by side effects, high costs, and frequent relapse [1]. Therefore, there is growing interest in plant-derived functional foods as complementary approaches for UC management, particularly those rich in bioactive compounds with anti-inflammatory and antioxidant properties [12,13,14]. Among these, polyphenols have attracted considerable attention due to their ability to modulate inflammatory responses and gut microbiota, highlighting their potential as promising dietary interventions for UC [15].

*Pyracantha fortuneana (Maxim.)* H.L.Li is an edible plant widely distributed in East Asia and Southern Europe, whose fruits are rich in polyphenols and other bioactive components [16,17]. Previous studies have demonstrated that *P. fortuneana* fruit extract (PFE) exhibits antioxidant and metabolic regulatory effects, including the improvement of intestinal barrier integrity and modulation of gut microbiota in metabolic disorder models [18,19]. However, its potential effects on colitis and the associated biological responses remain unclear.

We assessed whether PFE could limit colonic injury in mice exposed to DSS. To characterize the accompanying biological responses, we profiled bacterial communities by 16S rRNA sequencing, intestinal metabolites by untargeted metabolomics, and host gene expression by transcriptomic analysis. Together, these measurements allowed us to examine PFE-associated changes across microbial, metabolic, and host processes and explore the extract’s relevance to dietary approaches for intestinal health.

## 2. Materials and Methods

### 2.1. Chemicals and Reagents

The fruits of *Pyracantha fortuneana* (Maxim.) H.L.Li (PFF) were harvested from wild, mature plants in Jinsha County, Bijie City, Guizhou Province, China. The species was authenticated by Professor Maoteng Li (College of Life Science and Technology, Huazhong University of Science and Technology) based on the morphological characteristics of the leaves and fruits, with reference to a voucher specimen (No. GZTM0064659) held at Guiyang University of Traditional Chinese Medicine, as previously described [18]. Only fruits with fully red skin were selected, with ripeness assessed visually based on skin coloration. The fruits were subsequently washed and freeze-dried for further analysis. Dextran sulfate sodium (DSS) and 5-aminosalicylic acid (5-ASA) were obtained from MP Biomedicals (Irvine, CA, USA) and Bofei Yipusheng Pharmaceutical Co., Ltd. (Tianjin, China), respectively. The fecal occult blood detection kit was purchased from Beijing Solarbio Science & Technology Co., Ltd. (Beijing, China). Mouse ELISA kits for IL-10, DAO, and GSH-Px were supplied by Jiangsu Meimian Industrial Co., Ltd. (Yancheng, China), and those for IL-6 and TNF-α by Wuhan Servicebio Biotechnology Co., Ltd. (Wuhan, China). Physiological saline was obtained from Henan Kelun Pharmaceutical Co., Ltd. (Anyang, China). Wuhan Servicebio Biotechnology Co., Ltd. (Wuhan, China) supplied H&E staining solution, RNA extraction solution, reverse transcriptase, DNA polymerase I, and cDNA synthesis kits. TRIzol was obtained from Thermo Fisher Scientific (Waltham, MA, USA), and Western blot antibodies from Bioss Inc. (Woburn, MA, USA) and ABclonal Biotechnology Co., Ltd. (Beijing, China).

### 2.2. Preparation of PFE

PFE was prepared following the extraction method previously established by our research group, with slight modifications [20]. The extraction temperature of 55 °C was consistent with that used in our previous study on *P. fortuneana* fruit extraction [18,19]. Briefly, freeze-dried PFF powder was weighed and mixed with 70% ethanol at a solid-to-liquid ratio of 1:10 (*w*/*v*). The mixture was stirred thoroughly and allowed to macerate overnight. Subsequently, it was stirred using a magnetic stirrer (Zhengzhou Greatwall Scientific Industrial and Trade Co., Ltd., Zhengzhou, China) at 55 °C for 30 min, followed by ultrasonic extraction for an additional 30 min. The resultant solution was filtered through 250-mesh gauze, with this filtration process repeated three times. All collected supernatants were combined, concentrated using a RE-2000B rotary evaporator (Yuankai Instrument Equipment Co., Ltd., Shanghai, China), and freeze-dried for storage at −20 °C until further use.

### 2.3. Analytical Characterization of PFE

PFE was characterized by HPLC–UV and UPLC–ESI–QTRAP–MS/MS. For HPLC–UV, overnight ethanol precipitation of residual proteins and polysaccharides was followed by centrifugation at 6000× *g* for 15 min and filtration of the supernatant through a 0.22 μm membrane. An Agilent 1200 system with a UV detector and C18-aq column (250 mm × 4.6 mm, 5 μm; Agilent Technologies, Santa Clara, CA, USA) was used. Mobile phases were formic acid/acetonitrile (1:1000, *v*/*v*; A) and formic acid/water (1:100, *v*/*v*; B), with the following gradient: 0–15 min, 5–11% A; 15–25 min, 11% A; 25–40 min, 11–14% A; 40–45 min, 14–18% A; 45–53 min, 18–28% A; 53–70 min, 28–35% A; 70–85 min, 35–90% A; 85–90 min, 90–5% A; and 90–95 min, 5% A. The mixed mobile-phase formic acid concentration decreased as A increased and returned to its initial level during re-equilibration. The flow rate, injection volume, and detection wavelengths were 1.0 mL/min, 10 μL, and 280/325 nm, respectively. Compound identities were established against authentic standards using retention times and UV absorption characteristics; quantification used external calibration curves. Total phenolics were measured by the Folin–Ciocalteu method and expressed as gallic acid equivalents per gram of dry weight (mg GAE/g DW).

For UPLC–ESI–QTRAP–MS/MS profiling, freeze-dried PFE (50 mg) was extracted with 1.2 mL of 70% aqueous methanol containing internal standards and precooled to −20 °C. Samples were vortexed for 30 s at 30 min intervals (six cycles), centrifuged at 12,000 rpm for 3 min, and filtered through a 0.22 μm membrane. Metware Biotechnology Co., Ltd. (Wuhan, China) performed the analysis using an ExionLC™ AD system coupled to an ESI triple-quadrupole linear ion-trap mass spectrometer, both from SCIEX (Framingham, MA, USA). Separation used an Agilent SB-C18 column (1.8 μm, 2.1 mm × 100 mm) with water and acetonitrile, both containing 0.1% formic acid. Acetonitrile increased from 5% to 95% over 9 min, remained at 95% for 1 min, returned to 5% within 1.1 min, and remained at 5% until 14 min. Operating conditions were 0.35 mL/min, 40 °C, and a 2 μL injection. Positive- and negative-ion acquisition used a source temperature of 500 °C and ion-spray voltages of 5500 and −4500 V, respectively. Multiple reaction monitoring data were annotated against MWDB using retention times and precursor-to-product ion transitions.

### 2.4. Animals and Treatments

Male SPF-grade C57BL/6 mice, aged 6–8 weeks and weighing 20–23 g (Animal Quality Certificate No. 210726230101223726), were used in this study. All mice were housed under specific pathogen-free conditions at Huazhong University of Science and Technology at 25 ± 1 °C, 55–65% relative humidity, and a 12 h light/dark cycle. The experimental protocol was approved by the Institutional Animal Care and Use Committee of Huazhong University of Science and Technology (Approval No. HUST-IACUC-3905).

**Figure 1:**
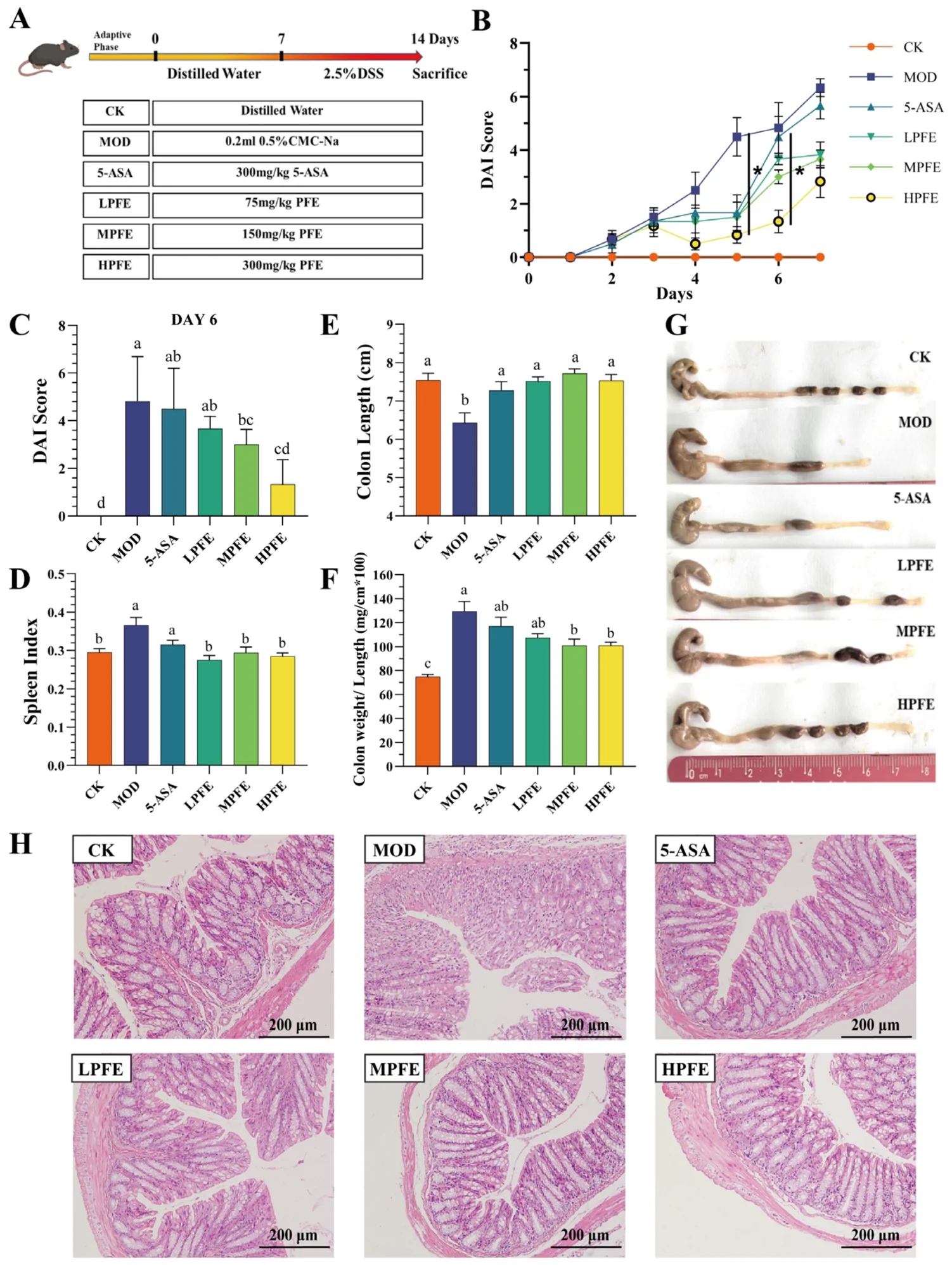
PFE ameliorated DSS-induced colitis symptoms. (**A**) Schematic representation of DSS-induced colitis and PFE treatment protocol. (**B**) Disease activity index (DAI) scores across experimental days. (**C**) DAI score comparison on Day 6. (**D**) Spleen index comparison between groups. (**E**) Colon length measurement. (**F**) Colon weight/length ratio. (**G**) Representative images of mouse colons. (**H**) Histological images of colon tissues stained with H&E. Groups not sharing the same lowercase letter differ significantly (*p* < 0.05). * *p* < 0.05 versus the MOD group.

After a 7-day acclimatization period, the mice were randomly assigned to six groups (*n* = 8 per group): control (CK), model (MOD), positive control (5-ASA), low-dose PFE (LPFE), medium-dose PFE (MPFE), and high-dose PFE (HPFE). PFE and 5-ASA were suspended in 0.5% (*w*/*v*) carboxymethyl cellulose sodium (CMC-Na) for oral administration. During the 7-day pretreatment period, the 5-ASA group received 5-ASA at 300 mg/kg/day, while the LPFE, MPFE, and HPFE groups received PFE at 75, 150, and 300 mg/kg/day, respectively. The CK group received an equivalent volume of distilled water, whereas the MOD group received an equivalent volume of 0.5% CMC-Na. During the subsequent 7-day modeling period, all groups except CK received 2.5% DSS in the drinking water, while the respective daily gavage treatments were continued unchanged. The experimental design is shown in Figure 1A.

### 2.5. Evaluation of Colitis Severity

Body weight, stool consistency, and fecal bleeding were assessed daily throughout the 7-day DSS challenge. For occult blood testing, fresh feces on a white porcelain plate received two drops each of O-tolidine solution and oxidizing solution; blue coloration within 2 min indicated a positive result. The disease activity index (DAI) was calculated following Dong et al. [21] by summing the scores for body weight loss, stool consistency, and fecal bleeding, using the criteria in Supplementary Table S1.

At the end of the experiment, the mice were fasted for 12 h and anesthetized. Blood samples were subsequently collected, after which the mice were euthanized by cervical dislocation under anesthesia. The blood samples were allowed to clot at 4 °C for 12 h and then centrifuged at 3000× *g* for 15 min. The resulting serum was collected and stored at −80 °C until analysis. The spleen was excised, gently dried, and weighed, and the spleen index was calculated as the ratio of spleen weight to body weight.

The colon was excised from the ileocecal junction to the distal rectum, and its length and weight were recorded. The colon weight-to-length ratio was calculated as an indicator of colonic edema. Cecal contents were aseptically collected in sterile tubes and stored at −80 °C for gut microbiota and metabolomics analyses. The colon was rinsed with prechilled phosphate-buffered saline and gently dried. A representative segment was fixed in 4% paraformaldehyde for histopathological and immunofluorescence analyses, while the remaining tissue was stored at −80 °C for transcriptomic and Western blot analyses.

### 2.6. Histopathological and Immunofluorescence Analyses

Histopathological and immunofluorescence staining of colon tissues was performed by Wuhan Servicebio Technology Co., Ltd. (Wuhan, China). Colon tissues fixed in 4% paraformaldehyde were embedded in paraffin and sectioned at a thickness of 4 μm. For histopathological examination, the sections were stained with hematoxylin and eosin (H&E), and representative images were acquired using a light microscope.

Immunofluorescence analysis included colon sections from six mice in each of the CK, MOD, and HPFE groups. Following deparaffinization, the sections received primary antibodies targeting Occludin and ZO-1, with incubation at 4 °C overnight. The corresponding Cy3-labeled secondary antibodies were then applied for 45 min under light-protected conditions, followed by DAPI counterstaining of the nuclei. Images obtained by fluorescence microscopy were analyzed in ImageJ (version 1.54j; National Institutes of Health, Bethesda, MD, USA) to quantify the relative fluorescence intensity of each target protein.

### 2.7. ELISA

ELISA measurements included TNF-α, IL-6, IL-10, and glutathione peroxidase (GSH-Px) in serum, as well as diamine oxidase (DAO) in colon tissue homogenates. Each analyte was assayed with its corresponding mouse ELISA kit under the conditions specified by the manufacturer. Analyte concentrations were obtained from the standard curve generated for each assay.

### 2.8. Gut Microbial Analysis

Cecal contents from the CK, MOD, and HPFE groups were thawed on ice. For the microbiota analysis, the HPFE group was designated as the PFE group. Microbial genomic DNA was isolated with the OMEGA Soil DNA Kit (D5625-01; Omega Bio-Tek, Norcross, GA, USA), following the supplier’s protocol. Amplification targeted the bacterial 16S rRNA V3-V4 region with the primer pair 338F (5′-ACTCCTACGGGAGGCAGCA-3′) and 806R (5′-GGACTACHVGGGTWTCTAAT-3′). After preparation and quality assessment, the sequencing libraries were run on an Illumina NovaSeq instrument (Illumina, Inc., San Diego, CA, USA). The NovaSeq 6000 SP Reagent Kit provided 500 cycles for paired-end reads of 250 bp in each direction.

Sequence data were processed in QIIME 2 (version 2019.4). Demultiplexing and primer removal were performed with the demux and cutadapt plugins, respectively, followed by DADA2-based filtering, denoising, paired-read merging, and chimera removal to obtain ASVs. Taxonomic annotation and subsequent analyses were conducted on the GenesCloud platform (Personalbio, Shanghai, China; https://www.genescloud.cn/). Simpson and Pielou’s evenness indices were used to describe α-diversity, while Bray–Curtis distances were ordinated by PCoA to examine differences in community structure. LEfSe analysis was performed on the GenesCloud platform to identify taxa differing in abundance among groups.

### 2.9. Non-Targeted Metabolomics Analysis of Cecal Contents

Metabolomic profiling was conducted on cecal contents from the CK, MOD, and HPFE groups (*n* = 6 per group), with HPFE denoted as PFE for this analysis. Twenty milligrams of each sample was mixed with 400 μL methanol/water (7:3, *v*/*v*) containing internal standards. After vortex mixing and sonication in an ice-water bath, extracts were kept at −20 °C for 30 min and centrifuged at 12,000 rpm for 10 min at 4 °C. The recovered supernatant underwent an additional 3-min centrifugation at 12,000 rpm, and a 200 μL aliquot was collected for LC–MS analysis. Chromatographic separation employed a Waters ACQUITY Premier HSS T3 column (1.8 μm, 2.1 mm × 100 mm; Waters Corporation, Milford, MA, USA) maintained at 40 °C. The aqueous and organic phases were 0.1% formic acid in water and 0.1% formic acid in acetonitrile, respectively; the gradient is given in Table S2. Samples (4 μL) were analyzed at a flow rate of 0.4 mL/min. An AB TripleTOF 6600 mass spectrometer (SCIEX, Framingham, MA, USA) operated under both positive- and negative-ion conditions was used for mass spectrometric acquisition, with the instrumental settings listed in Table S3. PCA was used to visualize overall metabolic variation, whereas OPLS-DA was used for supervised group discrimination and evaluated by 200 permutation tests. Differential features were selected using VIP > 1.0, FC > 1.2 or <0.8, and *p* < 0.05, followed by KEGG enrichment. Data processing and statistical analyses were performed through the Metware Cloud platform (https://cloud.metware.cn).

### 2.10. Transcriptomic Analysis of Colon Tissues

Colon tissues from CK, MOD, and HPFE mice (*n* = 3 per group) were selected for RNA-seq, with HPFE designated as PFE in the transcriptomic dataset. Total RNA was isolated with TRIzol. RNA quantity and integrity were evaluated using a Qubit fluorometer (Thermo Fisher Scientific, Waltham, MA, USA) and Qsep400 biofragment analyzer before library preparation. Sequencing was conducted by Metware Biotechnology Co., Ltd. (Wuhan, China) on an Illumina platform to obtain 150-bp paired-end reads. Following read-quality control, sequences were mapped against the Mus musculus genome assembly GRCm39 (Ensembl release 109). Gene counts obtained with FeatureCounts (version 2.0.3) were analyzed in DESeq2 (version 1.38.3). A gene was considered differentially expressed when |log2FC| ≥ 1, *p* < 0.05, and FDR < 0.05. Global variation among transcriptomes was examined by PCA, and KEGG enrichment was subsequently performed for the identified DEGs.

### 2.11. Western Blotting Analysis

Western blotting was conducted using colon samples from the CK, MOD, and HPFE groups (*n* = 3 per group); HPFE is referred to as PFE in this analysis. Colon tissue was homogenized in ice-cold RIPA buffer supplemented with PMSF. Lysates were clarified by centrifugation at 12,000 rpm for 5 min at 4 °C, and protein content in the recovered supernatant was determined by BCA assay. For each biological sample, 40 μg protein was resolved by SDS-PAGE and transferred to a PVDF membrane. After 2 h of blocking in TBST containing 5% non-fat milk, membranes were exposed at 4 °C overnight to antibodies against ASBT, PPARγ, NLRP6, or β-actin. HRP-conjugated secondary antibodies were applied for 2 h at room temperature after TBST washing. Chemiluminescent signals were then recorded, and band intensities were determined with Image-Pro Plus (version 6.0; Media Cybernetics, Rockville, MD, USA; https://www.mediacy.com/imagepro, accessed on 1 September 2026). ASBT, PPARγ, and NLRP6 abundance was expressed relative to β-actin.

### 2.12. Statistical Analysis

RNA-seq and Western blot analyses each used three independent biological replicates per group, derived from different mice. For these analyses, sample sizes were based on published studies; no a priori power analysis was performed. Statistical testing was carried out in GraphPad Prism 9.4.1 (GraphPad Software, San Diego, CA, USA). Body-weight and DAI trajectories were evaluated by two-way repeated-measures ANOVA with treatment and time as factors. One-way ANOVA was used for endpoint variables involving more than two groups, whereas two-group comparisons employed an unpaired two-tailed Student’s *t*-test. Tukey-adjusted multiple comparisons followed both ANOVA procedures where appropriate. Values are expressed as mean ± SEM, and statistical significance was defined as *p* < 0.05 using two-sided tests. Additional analytical procedures for the microbiota, metabolomic, and transcriptomic datasets are specified in their respective sections. Genus–metabolite relationships were explored by Spearman correlation using the 18 matched animals. To examine whether these associations persisted beyond group differences, group-adjusted rank correlations were additionally calculated. *p* values were obtained from 99,999 permutations, with permutations constrained within treatment groups for the adjusted analysis. Benjamini–Hochberg correction was performed separately for the 260 tests in each analysis, and *q* < 0.05 was used as the significance threshold.

## 3. Results

### 3.1. Chemical Characterization of PFE

The chemical profile of PFE was characterized using complementary HPLC and UPLC–MS/MS analyses. A representative HPLC–UV chromatogram with the peaks corresponding to the nine quantified phenolic compounds, together with UPLC–ESI–QTRAP–MS/MS profiles, is provided in Figure S1. As summarized in Table 1, nine representative phenolic compounds were identified and quantified by HPLC through comparison of their retention times and UV absorption characteristics with those of authentic standards. These compounds included the phenolic acids chlorogenic acid and cryptochlorogenic acid; the flavonoid-related compounds rutin, isoquercitrin, hyperoside, and epicatechin; and the condensed tannins procyanidins B1, B2, and B4. UPLC–ESI–QTRAP–MS/MS analysis provided complementary mass spectrometric information supporting the chemical profiling of the extract.

Among the quantified compounds, rutin was the most abundant constituent (5.118 mg/g DW), followed by isoquercitrin (2.401 mg/g DW), procyanidin B2 (1.261 mg/g DW), and procyanidin B4 (1.048 mg/g DW). Procyanidin B1, hyperoside, epicatechin, chlorogenic acid, and cryptochlorogenic acid were detected at comparatively lower concentrations. The total phenolic content of PFE was 65.53 mg GAE/g DW. Collectively, these findings confirm the phenolic-rich nature of PFE and establish its representative chemical composition for the subsequent in vivo evaluation.

### 3.2. PFE Alleviated DSS-Induced Colitis in Mice

To evaluate the protective effects of PFE against DSS-induced colitis, mice were orally administered different doses of PFE for 7 days before DSS exposure, and the treatments were continued throughout the subsequent 7-day DSS challenge (Figure 1A). DSS administration markedly increased the disease activity index (DAI), confirming the successful establishment of experimental colitis (Figure 1B). PFE treatment produced a dose-related reduction in DAI scores, with the HPFE group showing significantly lower scores than the MOD group on days 5 and 6 (*p* < 0.05). Consistently, the DAI score on day 6 was markedly reduced in the MPFE and HPFE groups (Figure 1C). DSS exposure also caused body weight loss relative to the CK group; however, PFE treatment did not significantly affect body weight changes during the experimental period (Figure S2).

DSS-treated mice exhibited a significantly increased spleen index, shortened colons, and an elevated colon weight-to-length ratio compared with the CK group (Figure 1D–G). PFE treatment significantly reduced the spleen index and restored colon length. The colon length decreased from 7.54 ± 0.18 cm in the CK group to 6.43 ± 0.26 cm in the MOD group but was restored following PFE administration (Figure 1E). PFE also reduced the DSS-induced increase in the colon weight-to-length ratio, with significant reductions observed in the MPFE and HPFE groups (Figure 1F).

Histopathological examination revealed a largely intact epithelial lining and well-organized crypts in the CK group (Figure 1H). In contrast, the MOD group showed epithelial disruption, disorganized crypt architecture with focal crypt loss, and marked inflammatory cell infiltration. Compared with the MOD group, the PFE-treated groups displayed better-preserved epithelial and crypt structures and less apparent inflammatory infiltration. The 5-ASA group also showed relatively preserved mucosal architecture. These qualitative histological observations, together with the clinical and macroscopic findings, support the protective effects of PFE against DSS-induced colonic injury.

### 3.3. PFE Modulated Inflammatory Cytokines and Serum GSH-Px Levels in DSS-Induced Colitis

**Figure 2:**
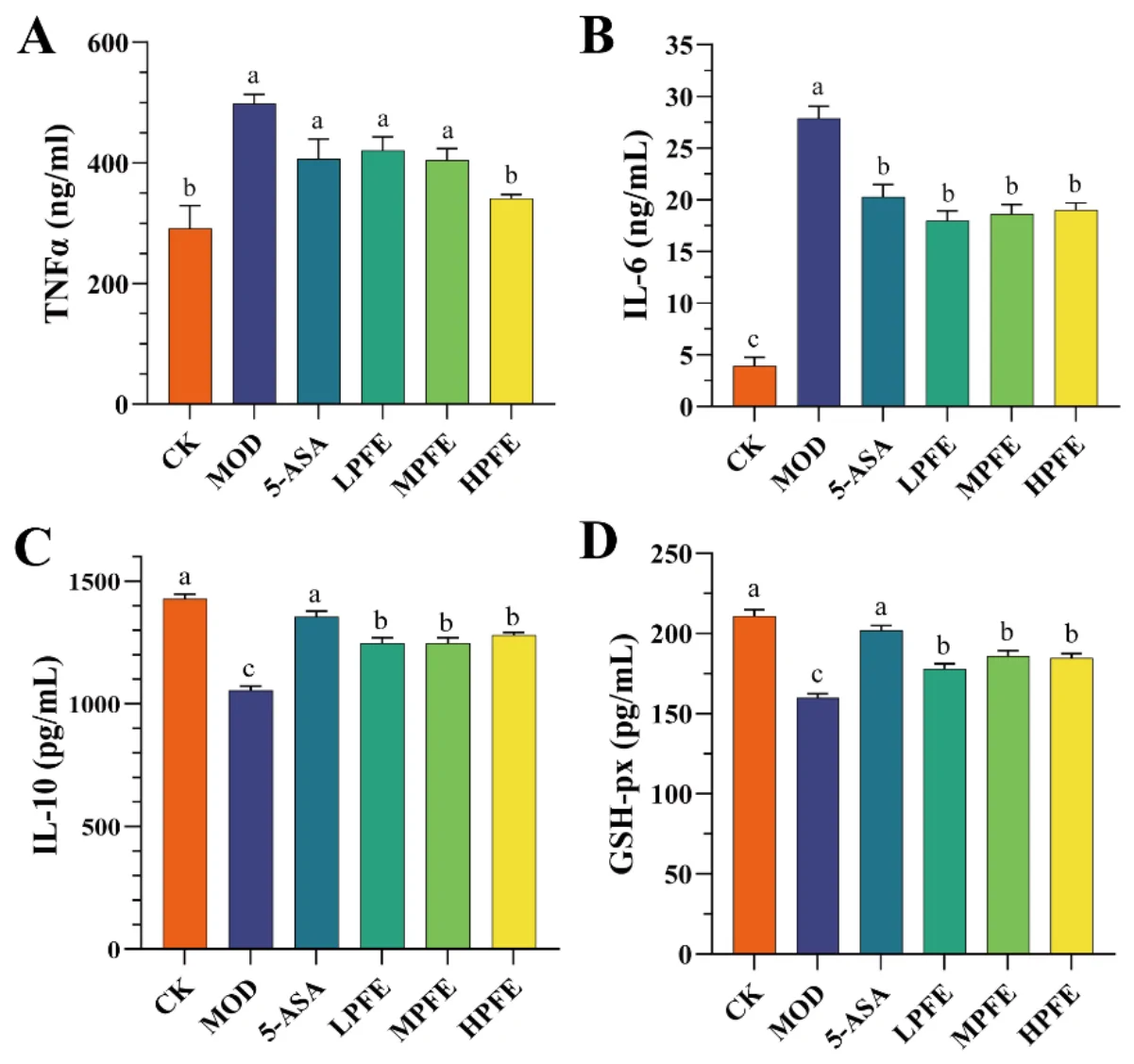
PFE reduced inflammation and oxidative stress in colitis mice. (**A**) TNF-α, (**B**) IL-6, and (**C**) IL-10 levels in serum. (**D**) GSH-Px concentration. Different letters (a, b, c) above bars indicate significant differences (*p* < 0.05).

Pro-inflammatory cytokines such as TNF-α and IL-6 play critical roles in the pathogenesis of ulcerative colitis, whereas IL-10 serves as an important anti-inflammatory mediator. Therefore, these cytokines were measured to evaluate the inflammatory status of mice with DSS-induced colitis. Serum measurements revealed an inflammatory shift following DSS exposure: TNF-α and IL-6 were significantly elevated, whereas IL-10 was significantly lower than in CK mice (Figure 2A–C). Increasing PFE doses were accompanied by a downward trend in TNF-α, but a statistically significant difference from the MOD group was detected only at the highest dose (Figure 2A). In contrast, all three doses of PFE significantly attenuated the DSS-induced increase in IL-6, with no significant differences observed among the PFE groups (Figure 2B). The reduction in IL-10 induced by DSS was also partially reversed by all PFE treatments. Although this effect was less pronounced than that of 5-ASA, each PFE-treated group exhibited a significantly higher IL-10 level than the MOD group (Figure 2C). Serum GSH-Px concentrations were significantly lower in MOD mice than in CK mice. Each PFE dose produced a significant increase relative to MOD, although concentrations in all three PFE groups remained below those observed in CK and 5-ASA mice (Figure 2D). Together, these results indicate that PFE modulated inflammatory cytokine profiles and partially restored serum GSH-Px levels in mice with DSS-induced colitis.

### 3.4. PFE Preserved Intestinal Barrier Integrity in DSS-Induced Colitis

**Figure 3:**
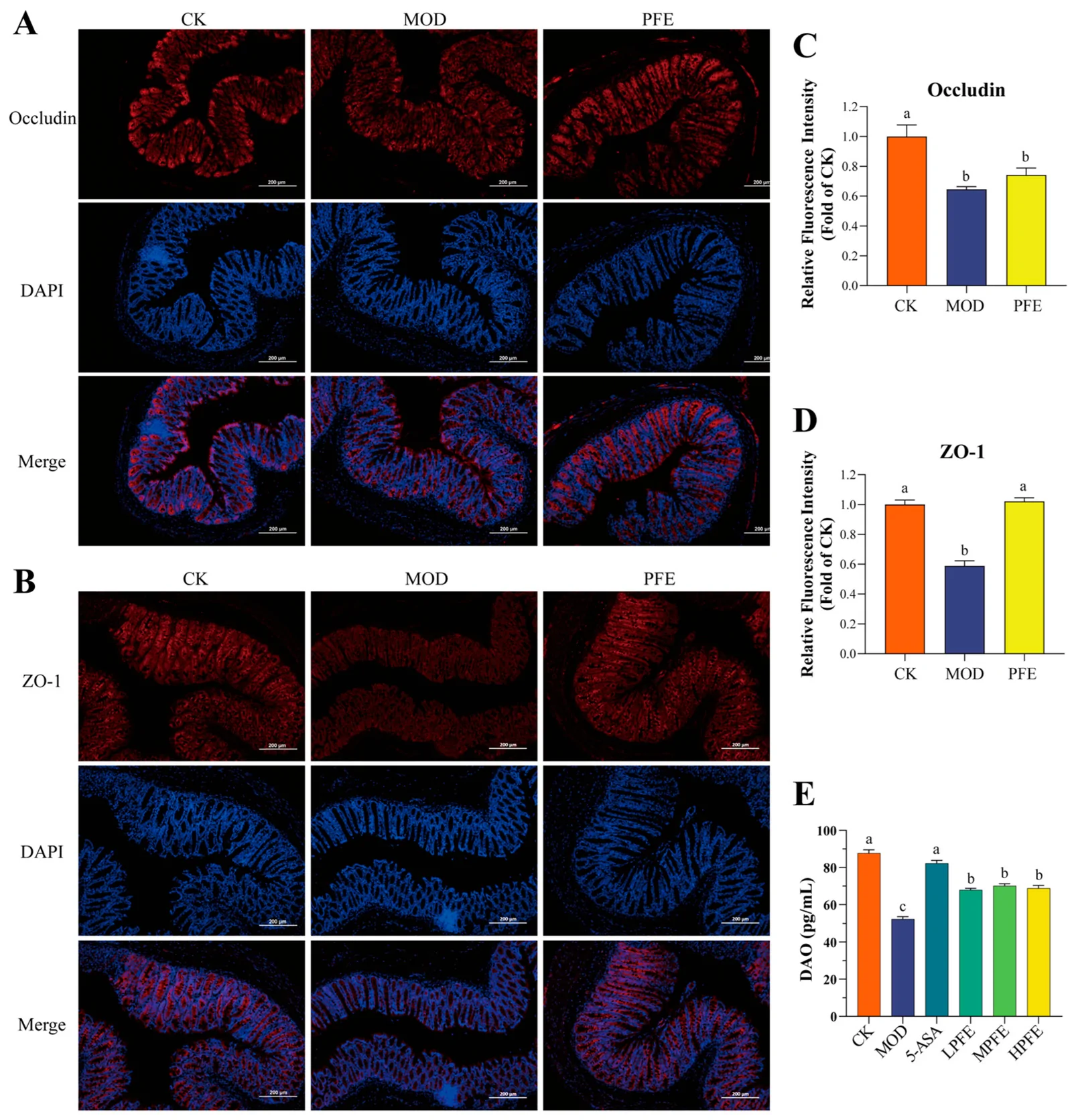
Effects of PFE on intestinal barrier-related parameters in mice with DSS-induced colitis. Representative immunofluorescence images of (**A**) Occludin and (**B**) ZO-1 in colon tissues. Target proteins are shown in red, and nuclei stained with DAPI are shown in blue. Scale bar: 200 μm. Relative fluorescence intensities of (**C**) Occludin and (**D**) ZO-1. (**E**) DAO levels in colon tissue homogenates. Data are presented as the mean ± SEM. Groups not sharing the same lowercase letter differ significantly (*p* < 0.05).

Tight junction proteins help preserve epithelial barrier integrity, which is often compromised during intestinal inflammation. We therefore used immunofluorescence to compare colonic expression of Occludin and ZO-1 among the CK, MOD, and HPFE groups. Representative immunofluorescence images showed red staining for Occludin and ZO-1 along the colonic mucosal epithelium, with nuclei counterstained blue using DAPI (Figure 3A,B). Compared with the CK group, the MOD group showed weaker target-protein fluorescence, particularly for ZO-1. Quantitative analysis confirmed that DSS treatment significantly reduced the relative fluorescence intensities of both proteins (Figure 3C,D). HPFE treatment increased Occludin fluorescence intensity numerically, although the difference from the MOD group was not statistically significant (Figure 3C). In contrast, ZO-1 staining was more prominent following HPFE treatment, consistent with a significant increase in its relative fluorescence intensity compared with the MOD group. No significant difference in ZO-1 fluorescence intensity was detected between the HPFE and CK groups (Figure 3D). DAO levels in colon tissue homogenates were further measured as a barrier-related biochemical parameter. The MOD group exhibited the lowest DAO level (52.23 ± 1.27 pg/mL). All three doses of PFE significantly increased DAO levels compared with the MOD group, with no significant differences among the PFE-treated groups (Figure 3E). However, DAO levels in these groups remained lower than those in the CK and 5-ASA groups. Together, these findings indicate that PFE partially restored tight junction protein expression and improved barrier-related parameters in mice with DSS-induced colitis.

### 3.5. PFE Modulated Gut Microbiota Composition in DSS-Induced Colitis

**Figure 4:**
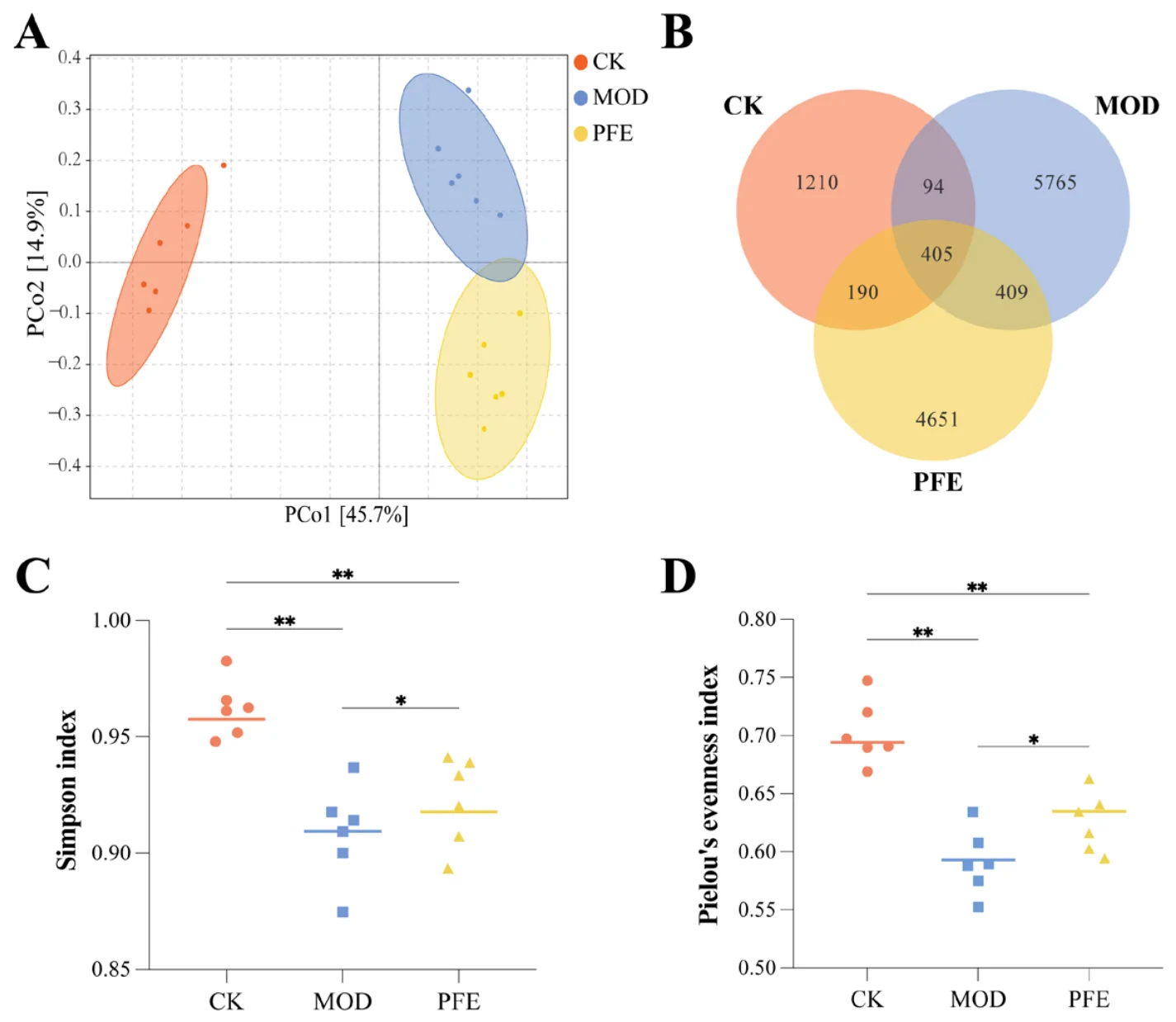
Effects of PFE on gut microbial diversity in mice with DSS-induced colitis. (**A**) Principal coordinate analysis (PCoA) based on Bray–Curtis dissimilarities. (**B**) Venn diagram showing shared and group-specific amplicon sequence variants (ASVs). (**C**) Simpson diversity index and (**D**) Pielou’s evenness index. The PFE group represents mice treated with high-dose PFE. Each point represents one mouse, and horizontal lines indicate group means (*n* = 6 per group). * *p* < 0.05, ** *p* < 0.01.

To investigate the effects of PFE on gut microbial composition, 16S rRNA gene sequencing was performed using cecal contents from the CK, MOD, and PFE groups. Principal coordinate analysis (PCoA) based on Bray–Curtis dissimilarities revealed clear separation among the three groups, indicating that DSS exposure and PFE treatment were associated with distinct microbial community structures (Figure 4A). The Venn diagram showed that 405 ASVs were shared among the three groups, whereas 1210, 5765, and 4651 ASVs were unique to the CK, MOD, and PFE groups, respectively (Figure 4B). Alpha-diversity analysis showed that DSS treatment significantly decreased the Simpson and Pielou’s evenness indices compared with the CK group, indicating reduced microbial diversity and evenness (Figure 4C,D). PFE treatment significantly increased both indices relative to the MOD group, although they remained lower than those in the CK group. These results suggest that PFE partially alleviated the loss of microbial diversity induced by DSS.

**Figure 5:**
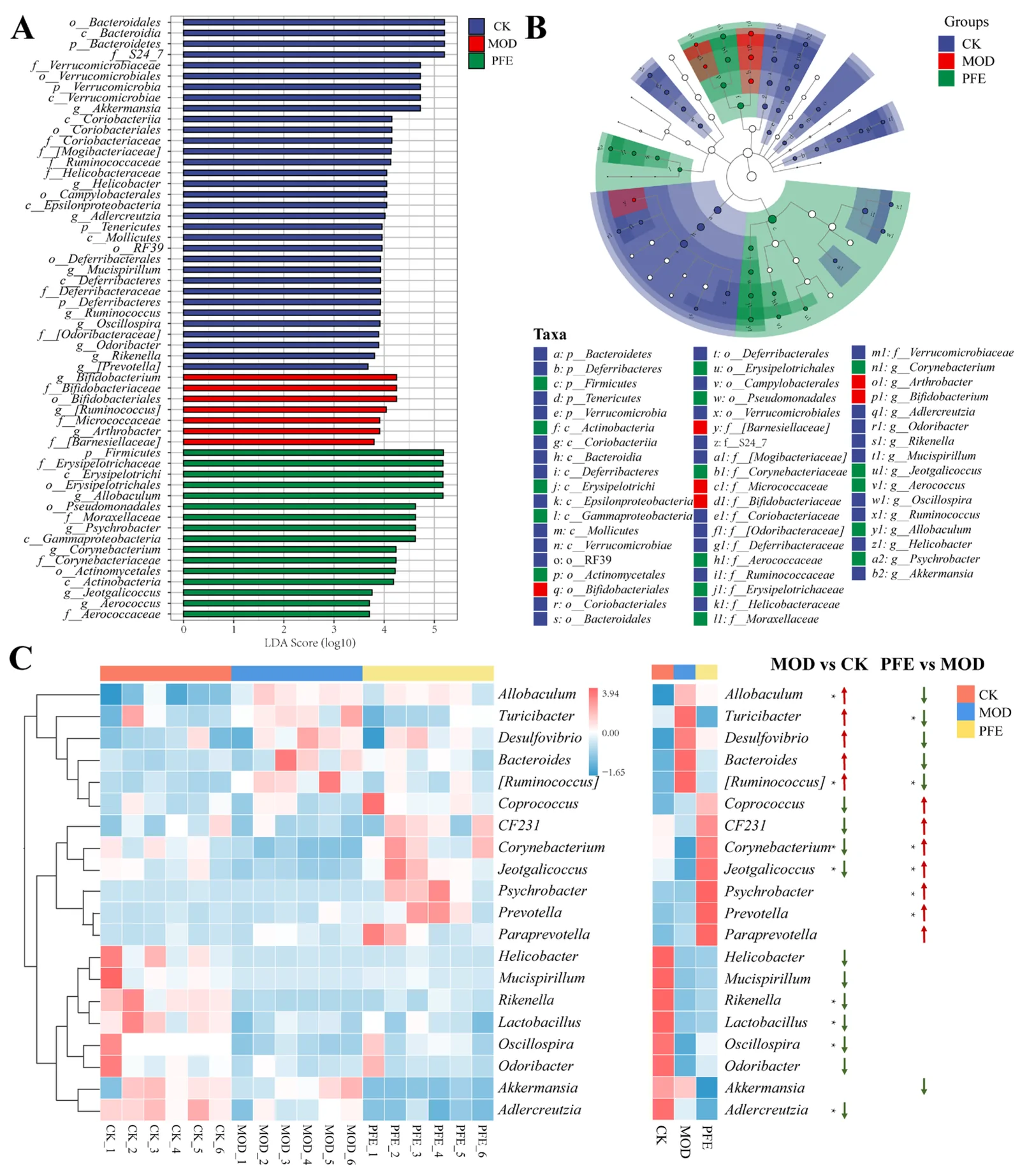
Effects of PFE on gut microbial taxonomic profiles in mice with DSS-induced colitis. (**A**) Linear discriminant analysis (LDA) scores of differentially abundant taxa identified by LEfSe. (**B**) LEfSe cladogram showing taxonomic differences among groups. (**C**) Heatmap and between-group comparisons of bacterial genera. Red upward arrows and green downward arrows indicate higher and lower relative abundances, respectively, in the first group relative to the second group in each labelled comparison; * indicates a significant difference between the corresponding groups (*p* < 0.05). The PFE group represents mice treated with high-dose PFE (*n* = 6 per group).

LEfSe analysis identified multiple taxa that were differentially enriched among the CK, MOD, and PFE groups, further demonstrating distinct taxonomic profiles across the treatments (Figure 5A,B). At the genus level, the MOD group showed higher relative abundances of *Allobaculum*, *Turicibacter*, *Desulfovibrio*, *Bacteroides*, and [*Ruminococcus*], together with lower relative abundances of *Lactobacillus*, *Odoribacter*, *Oscillospira*, *Rikenella*, and several other genera compared with the CK group (Figure 5C). Compared with the MOD group, PFE treatment significantly decreased the relative abundances of *Turicibacter* and [*Ruminococcus*] while significantly increasing those of *Corynebacterium*, *Jeotgalicoccus*, *Psychrobacter*, and *Prevotella*. In addition, *Coprococcus*, *CF231*, and *Paraprevotella* showed increasing trends following PFE treatment. Collectively, these findings indicate that PFE reshaped the DSS-altered gut microbial community and partially restored microbial diversity and evenness.

### 3.6. PFE Modulated Intestinal Metabolite Profiles in DSS-Induced Colitis

**Figure 6:**
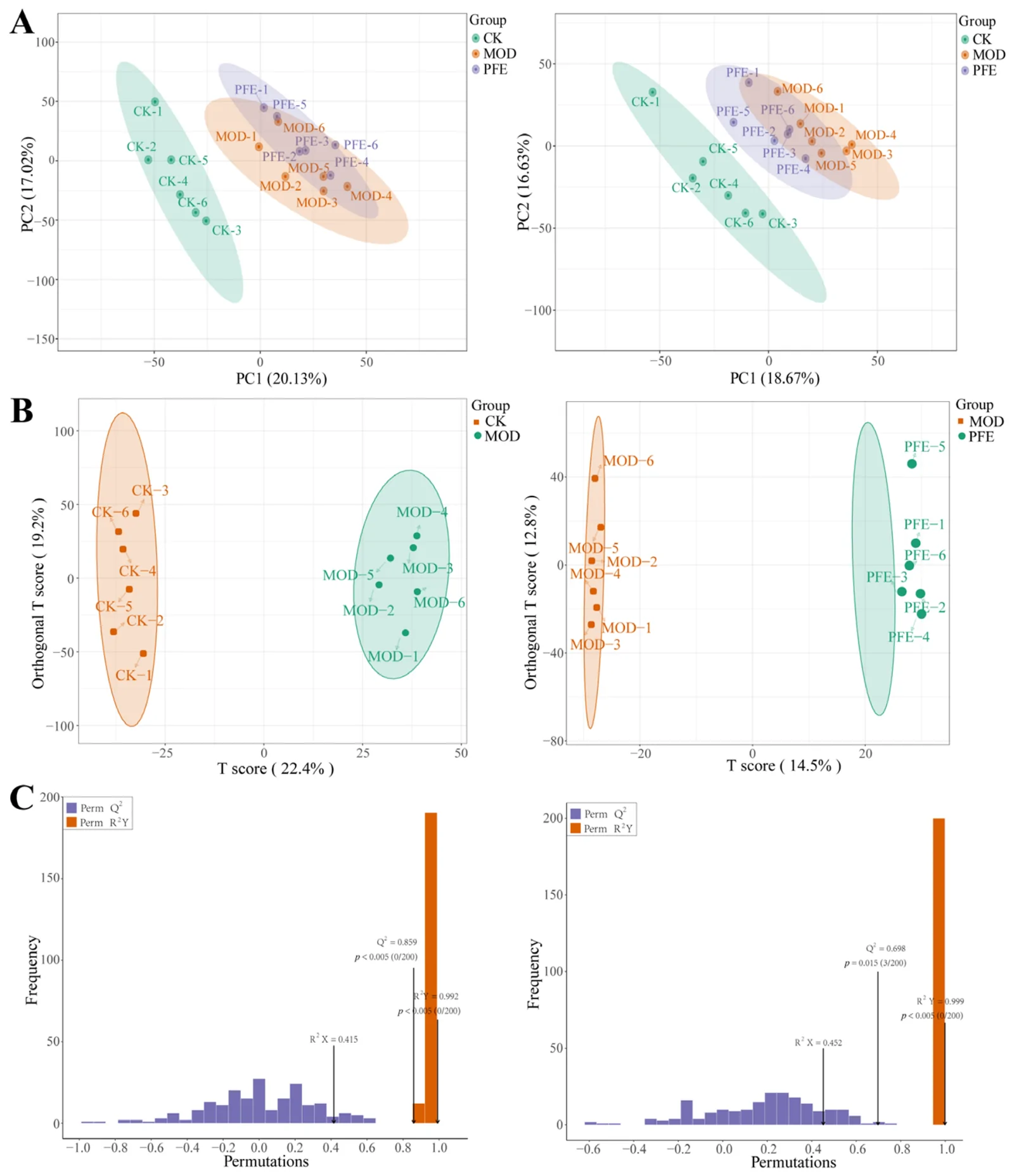
Multivariate analysis of cecal metabolite profiles. (**A**) PCA score plots in positive-ion (**left**) and negative-ion (**right**) modes. (**B**) OPLS-DA score plots for CK vs. MOD (**left**) and MOD vs. PFE (**right**). (**C**) Corresponding 200-permutation validation tests for CK vs. MOD (left) and MOD vs. PFE (right). PFE denotes the high-dose treatment group.

Non-targeted metabolomics was performed to characterize the effects of PFE on the metabolic profiles of cecal contents. A total of 7287 ion features were detected by combining the positive- and negative-ion datasets, from which 1466 metabolites were putatively annotated based on comprehensive mass spectrometric information. PCA showed clear separation of the CK group from the DSS-treated groups in both ionization modes, indicating marked metabolic alterations following DSS exposure (Figure 6A). Although the MOD and PFE groups partially overlapped, differences in their overall metabolic profiles were observed. OPLS-DA further demonstrated separation between the CK and MOD groups and between the MOD and PFE groups (Figure 6B). The CK vs. MOD yielded an R^2^Y value of 0.992 and a Q^2^ value of 0.859, whereas the MOD vs. PFE model yielded an R^2^Y value of 0.999 and a Q^2^ value of 0.698. The corresponding 200-permutation tests supported the validity of both models (Figure 6C).

**Figure 7:**
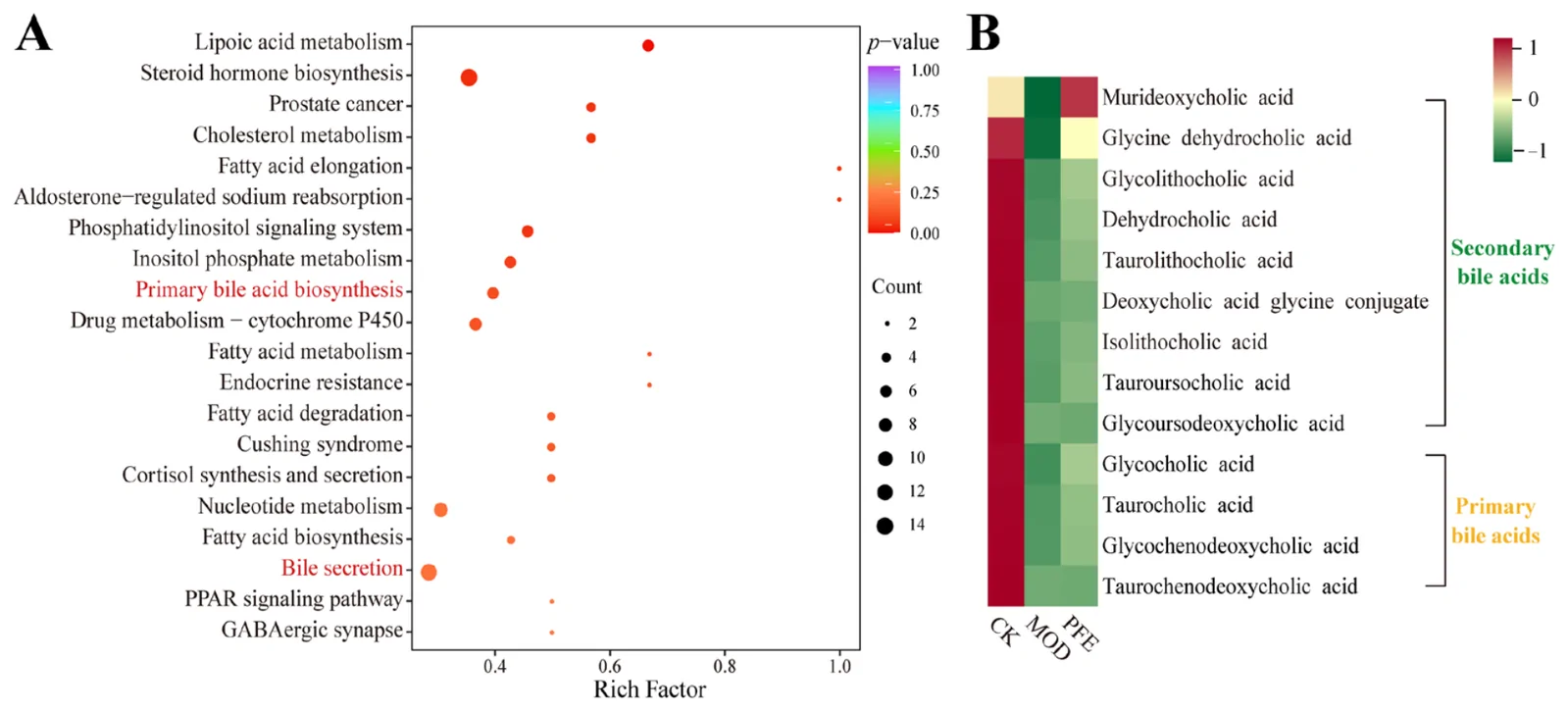
KEGG pathway enrichment and bile acid-related metabolite profiles. (**A**) KEGG enrichment analysis of differential metabolites; bubble size and colour indicate metabolite count and *p*-value, respectively. Pathway names highlighted in red indicate bile acid-related pathways emphasized in this study. (**B**) Heatmap of putatively annotated bile acid-related metabolites.

Based on the predefined screening criteria, 65 differential metabolites were identified between the CK and MOD groups, and 48 were identified between the MOD and PFE groups. Among these, 18 metabolites were shared between the two comparisons (Figure S3). KEGG enrichment analysis highlighted several pathways associated with lipid and bile acid metabolism, including lipoic acid metabolism, steroid hormone biosynthesis, cholesterol metabolism, fatty acid metabolism, primary bile acid biosynthesis, bile secretion, and the PPAR signaling pathway (Figure 7A). The bile acid heatmap showed that DSS treatment reduced the relative levels of multiple primary and secondary bile acids, whereas PFE partially reversed these alterations (Figure 7B). In particular, murideoxycholic acid, glycine dehydrocholic acid, and dehydrocholic acid showed higher relative levels in the PFE group than in the MOD group. Changes in several long-chain fatty acid- and acyl-related metabolites were also observed among the shared differential metabolites (Figure S3). Collectively, these results indicate that PFE modulated DSS-altered cecal metabolite profiles, particularly those associated with bile acid and lipid metabolism.

Exploratory analysis identified 15 significant genus–metabolite correlations (*q* < 0.05; Figure S4). Murideoxycholic acid was positively correlated with *Corynebacterium*, *Jeotgalicoccus*, and *Psychrobacter*, while glycine dehydrocholic acid was positively correlated with *Oscillospira*. Dehydrocholic acid was positively correlated with *Lactobacillus* and *Oscillospira* and negatively correlated with *Allobaculum* and [*Ruminococcus*]. However, no associations remained significant after group adjustment and multiple-testing correction, suggesting that the pooled correlations may partly reflect treatment-group differences.

### 3.7. PFE Modulated Colonic Transcriptional Profiles in DSS-Induced Colitis

**Figure 8:**
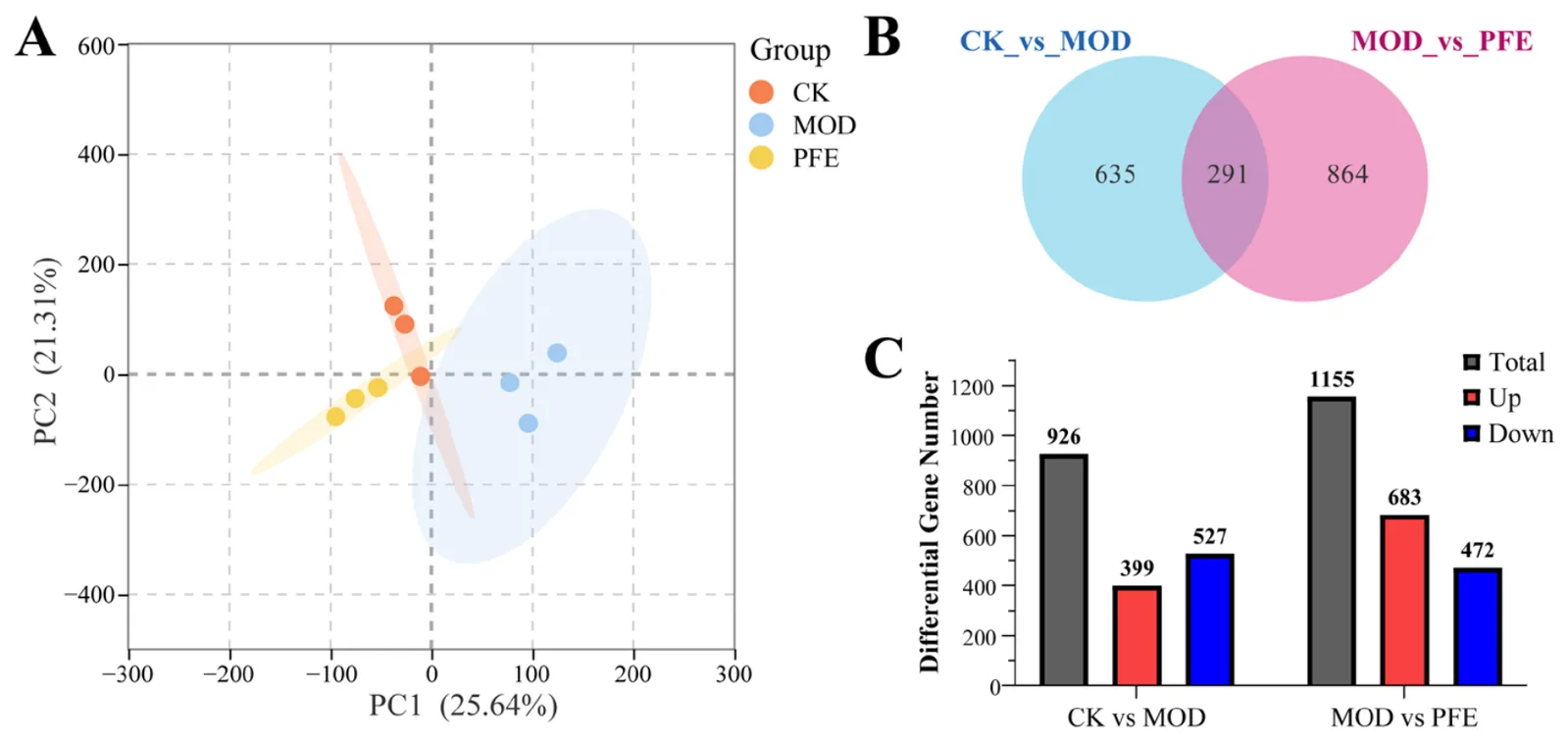
Effects of PFE on colonic transcriptomic profiles in mice with DSS-induced colitis. (**A**) PCA score plot. (**B**) Venn diagram showing shared and comparison-specific differentially expressed genes (DEGs). (**C**) Numbers of total, upregulated, and downregulated DEGs in each comparison.

Transcriptomic analysis was performed to characterize the colonic gene expression changes associated with PFE treatment. PCA showed distinct clustering of the CK, MOD, and PFE groups (Figure 8A). The MOD group was clearly separated from the CK group, whereas the PFE group exhibited a transcriptional profile closer to that of the CK group, indicating that PFE partially shifted DSS-altered gene expression patterns. The CK–MOD comparison yielded 926 DEGs (399 upregulated and 527 downregulated), whereas the MOD–PFE comparison yielded 1155 DEGs (683 upregulated and 472 downregulated; Figure 8C). The two DEG sets overlapped by 291 genes (Figure 8B), indicating that these genes differed in expression in both comparisons.

**Figure 9:**
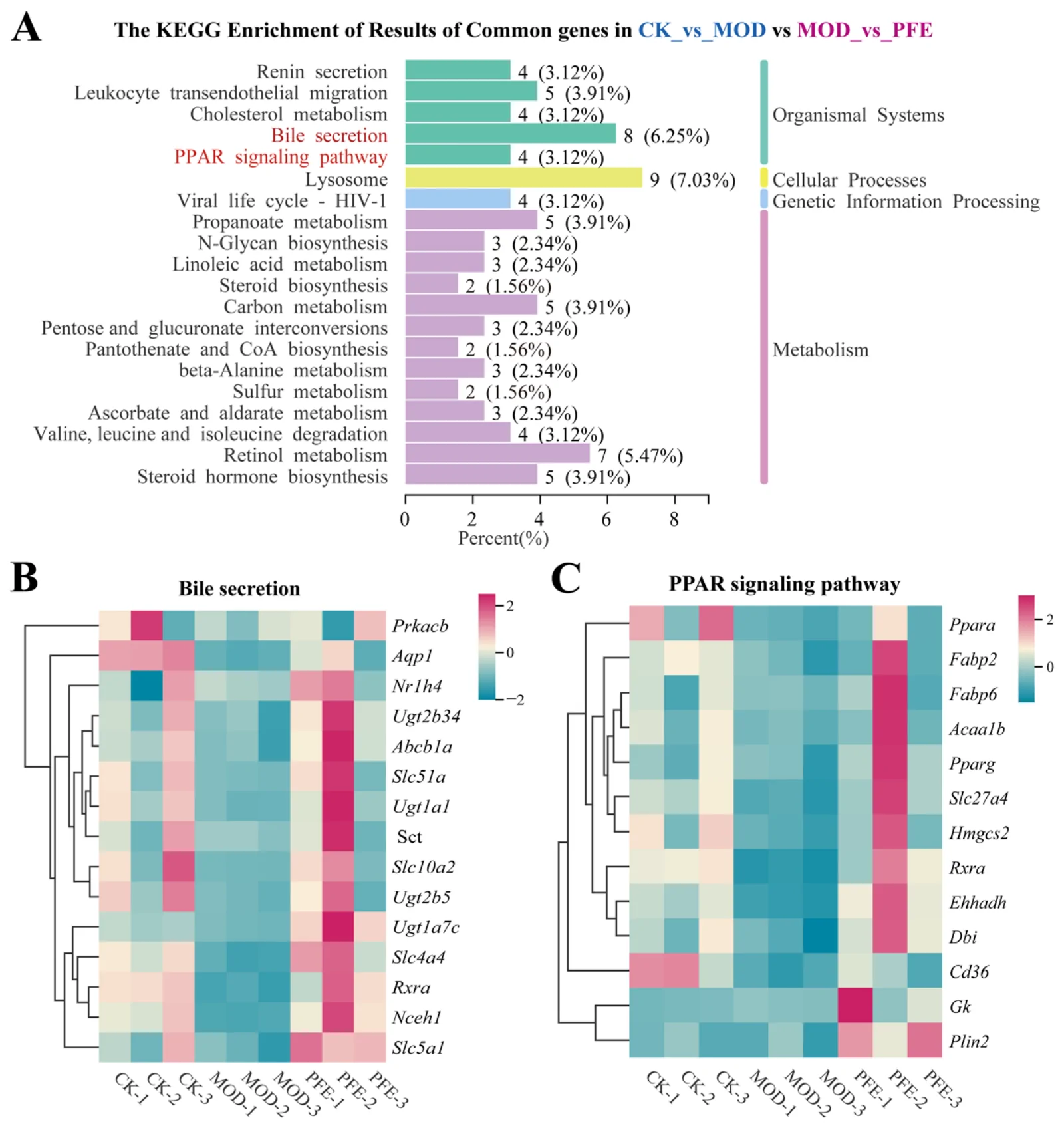
Pathway analysis of shared DEGs and expression profiles of selected pathway-related genes. (**A**) KEGG pathway analysis of DEGs shared between the CK–MOD and MOD–PFE comparisons. Numbers beside the bars indicate gene counts and percentages. Pathway names highlighted in red indicate the pathways selected for further expression profiling in panels B and C. (**B**,**C**) Heatmaps showing relative expression patterns of genes associated with bile secretion (**B**) and the PPAR signaling pathway (**C**).

KEGG enrichment analysis of the shared DEGs identified several pathways related to intestinal and metabolic functions, including bile secretion, the PPAR signaling pathway, lysosome, cholesterol metabolism, retinol metabolism, and steroid hormone biosynthesis (Figure 9A). Heatmap analysis further showed that PFE treatment altered the expression patterns of genes involved in bile secretion and bile acid transport, including *Nr1h4*, *Slc10a2*, *Slc51a*, and *Rxra* (Figure 9B). Genes associated with the PPAR signaling pathway and lipid metabolism, including *Pparg*, *Ppara*, *Fabp6*, *Acaa1b*, *Hmgcs2*, and *Cd36*, were also differentially expressed following PFE treatment (Figure 9C). These transcriptional changes were consistent with the metabolomic alterations in bile acid- and lipid-related pathways, suggesting that PFE was associated with coordinated changes in colonic metabolic regulation.

### 3.8. PFE Was Associated with Changes in ASBT/PPARγ/NLRP6-Related Protein Expression

**Figure 10:**
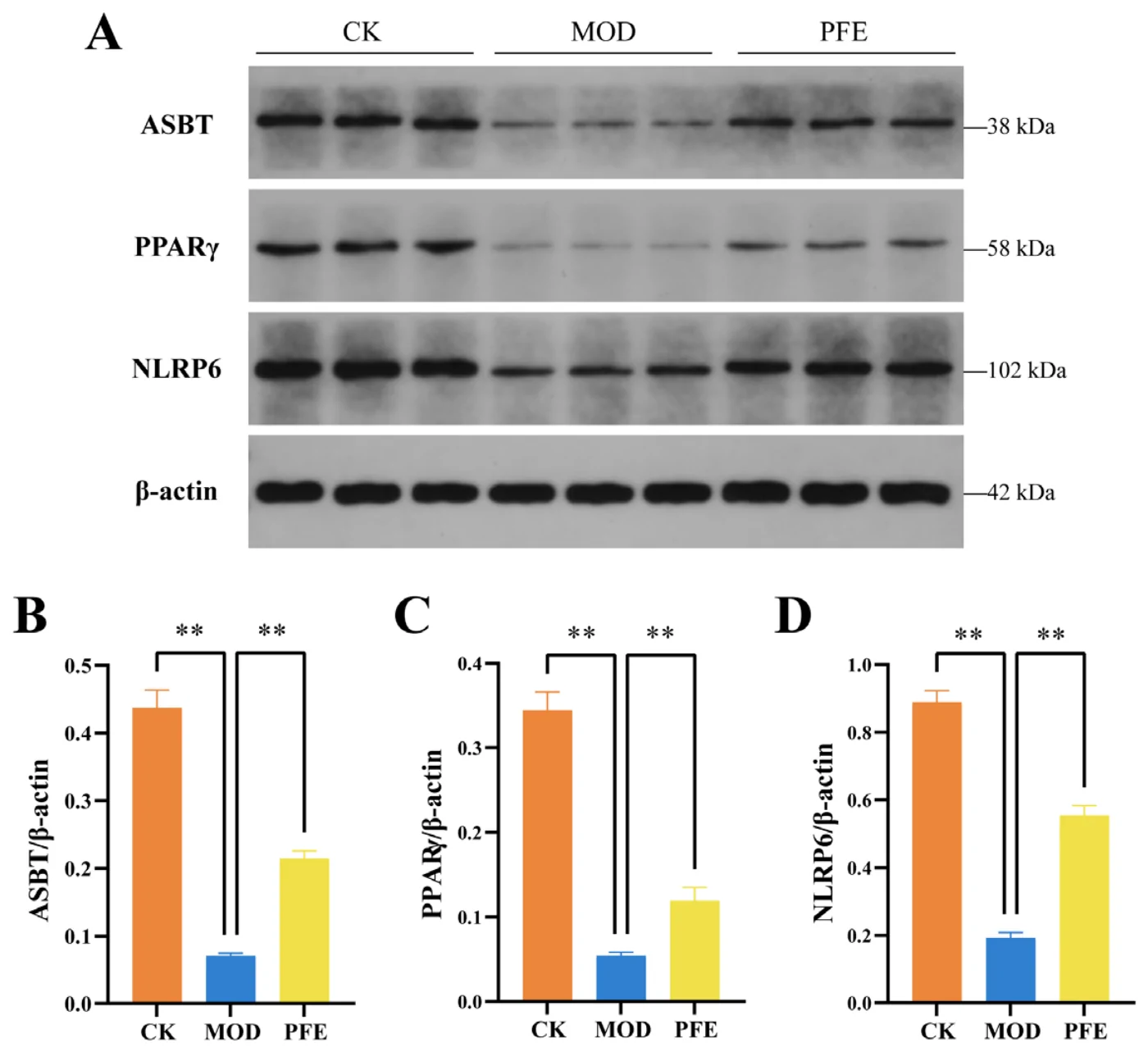
Effects of PFE on ASBT, PPARγ, and NLRP6 protein expression in the colons of mice with DSS-induced colitis. (**A**) Western blot profiles of ASBT, PPARγ, NLRP6, and β-actin. Relative band intensities of (**B**) ASBT, (**C**) PPARγ, and (**D**) NLRP6, with β-actin used for normalization. ** *p* < 0.01.

Protein levels of ASBT, PPARγ, and NLRP6 were further examined by Western blotting. As shown in Figure 10, DSS treatment significantly reduced the colonic protein levels of ASBT, PPARγ, and NLRP6 compared with the CK group. PFE treatment significantly increased the expression of all three proteins relative to the MOD group, although their levels remained lower than those in the CK group. ASBT expression was markedly decreased following DSS exposure and was partially recovered by PFE treatment (Figure 10A,B), consistent with the transcriptomic changes in genes associated with bile acid transport. PPARγ protein expression in the MOD group was reduced to approximately 15.7% of that in the CK group, whereas PFE treatment significantly increased its expression (Figure 10A,C). Similarly, NLRP6 expression was significantly decreased in the MOD group but increased by approximately 2.89-fold following PFE treatment (Figure 10A,D). Consistent with the protein-level change, *Nlrp6* transcript abundance was higher in the PFE group than in the MOD group (log_2_ fold change = 0.654; adjusted *p* = 0.024), although it did not meet the prespecified fold-change threshold for DEG classification. These findings indicate that PFE treatment was associated with changes in proteins involved in bile acid transport, PPAR signaling, and intestinal inflammatory regulation.

## 4. Discussion

The relapsing course of UC and its associated risk of colorectal cancer impose a substantial burden on patients’ quality of life. Concerns about adverse effects during prolonged pharmacological treatment have encouraged investigation of dietary and plant-derived bioactive compounds as complementary approaches [14,22,23]. *P. fortuneana* fruit has a long history of use in traditional medicine and as a dietary resource in China. Previous studies have demonstrated that its bioactive components, particularly polyphenols, exhibit anti-inflammatory and antioxidant activities and may contribute to intestinal barrier protection [19]. However, its potential role in colitis and the associated biological responses have not been systematically investigated. In the present study, chemical characterization confirmed the presence of representative phenolic acids, flavonoids, and condensed tannins in PFE, with rutin being the most abundant among the quantified compounds. However, its abundance alone does not establish that rutin was the principal contributor to the effects of PFE. As no single-compound controls were included, the observed effects relate to the whole extract; the contributions of individual constituents and possible additive or synergistic interactions require further investigation. We subsequently evaluated the protective effects of PFE in a DSS-induced colitis model and characterized the associated changes in gut microbiota, cecal metabolites, and colonic gene expression. Plant-derived microRNAs have been investigated as potential mediators of cross-kingdom gene regulation [24], with experimental studies reporting biological effects of MIR156a and miR160b-5p in human cell models [25,26]. These findings suggest that microRNAs may represent an additional class of bioactive components in plant preparations. However, their presence and activity in PFE were not assessed, and any contribution to its protective effects remains to be established.

The present results showed that PFE alleviated several clinical and pathological manifestations of DSS-induced colitis, as reflected by reduced DAI scores and spleen enlargement, attenuated colon shortening, and improved histopathological appearance. PFE also modulated the inflammatory response. In particular, the high-dose PFE treatment significantly reduced TNF-α levels, whereas all PFE doses decreased IL-6 and increased IL-10 levels compared with the MOD group. In addition, PFE increased serum GSH-Px levels, suggesting a partial improvement in the oxidative stress-related response induced by DSS. Because intestinal barrier disruption is a major feature of UC and is closely associated with mucosal inflammation [6], we further evaluated several barrier-related parameters. High-dose PFE significantly restored ZO-1 expression and produced a numerical, although nonsignificant, increase in Occludin expression. Moreover, all PFE treatments increased colonic DAO levels compared with the MOD group. Collectively, these changes suggest that the protective effects of PFE were associated with the modulation of inflammatory, oxidative stress-related, and intestinal barrier-related responses.

Alterations in gut microbiota are widely recognized in UC and are closely associated with disease progression [27,28,29]. In this study, DSS treatment markedly altered microbial community structure and reduced microbial diversity and evenness. PFE significantly increased the Simpson and Pielou indices compared with the MOD group, although these indices remained lower than those in the CK group. Moreover, the PCoA results indicated that PFE produced a distinct shift in microbial composition relative to the MOD group rather than complete restoration toward the CK group. At the genus level, PFE significantly decreased the relative abundances of [*Ruminococcus*] and *Turicibacter*, which have previously been associated with inflammatory conditions [30,31]. In contrast, the relative abundances of *Corynebacterium*, *Jeotgalicoccus*, *Psychrobacter*, and *Prevotella* were increased following PFE treatment. *Jeotgalicoccus* has been associated with lipid and carbohydrate metabolism, although its role in colitis remains unclear because inconsistent findings have been reported [28,32,33]. Furthermore, several genera including *Psychrobacter*, *Prevotella*, and *Paraprevotella* were increased following PFE treatment. Notably, *Prevotella* and *Paraprevotella* are often associated with polyphenol- and fiber-rich diets and have been linked to oxidative stress resistance and metabolic regulation [34]. *Coprococcus* has previously been reported to be reduced in IBD and associated with polyphenol-mediated microbiota modulation [35]. Interestingly, *Akkermansia* remained decreased in both the MOD and PFE groups, differing from our previous findings in a metabolic disorder model [19]. This discrepancy may reflect differences in disease context, inflammatory status, or treatment conditions. Collectively, these results suggest that PFE was associated with partial improvement in microbial diversity and distinct compositional changes in DSS-induced colitis, although the functional contributions of the affected taxa require further investigation.

Metabolites present in the intestinal lumen represent an important interface between the gut microbiota and host physiology [9]. In the present study, non-targeted metabolomic analysis of cecal contents revealed that DSS exposure markedly altered the overall metabolic profile, particularly pathways related to lipid metabolism, bile acid biosynthesis, and bile secretion. Among the putatively annotated metabolites, PFE treatment was associated with higher relative levels of several bile acid-related metabolites, particularly murideoxycholic acid, glycine dehydrocholic acid, and dehydrocholic acid, compared with the MOD group. Alterations in secondary bile acid profiles have frequently been observed in UC and have been associated with microbial dysbiosis and impaired intestinal homeostasis [8,36,37,38]. Moreover, secondary bile acids can participate in the regulation of intestinal immune responses and epithelial function [11]. Several secondary bile acids altered by PFE treatment have been reported to play important roles in host metabolic and inflammatory regulation. Dehydrocholic acid and its conjugated derivatives have also been associated with bile secretion and lipid digestion [39]. In addition, murideoxycholic acid and glycolithocholic acid have been reported to decrease under pathological conditions and are considered potential indicators of metabolic or inflammatory disturbances [40]. Nevertheless, because the present findings were derived from non-targeted metabolite annotation and relative abundance analysis, these changes should be interpreted as PFE-associated shifts in the cecal bile acid profile rather than definitive restoration of bile acid homeostasis. Changes in several long-chain fatty acid- and acyl-related metabolites were also observed among the shared differential metabolites. Some fatty acids can act as endogenous ligands or metabolic regulators of nuclear receptors, including PPARs, which play important roles in intestinal metabolic homeostasis and inflammatory regulation [41,42]. Therefore, the observed changes in bile acid- and lipid-related metabolites may be associated with the host transcriptional responses identified in this study, although their direct regulatory effects require further validation.

Transcriptomic analysis further revealed that PFE treatment was associated with changes in genes involved in bile acid transport and lipid metabolism. Several bile secretion-related genes, including *Slc10a2*, *Slc51a*, and *Fabp6*, were downregulated in DSS-treated mice and partially increased following PFE intervention. These transcriptional changes occurred in parallel with the alterations in bile acid-related metabolites, although a direct regulatory relationship between them cannot be established from the present data. Genes involved in lipid metabolism and PPAR signaling were also enriched among the shared DEGs. PPARγ has been widely reported to regulate intestinal inflammation and maintain epithelial homeostasis [42,43]. In the present study, DSS exposure reduced PPARγ expression, whereas PFE treatment significantly increased its expression at the protein level, consistent with the transcriptomic findings. NLRP6 was examined because of its reported association with intestinal permeability and inflammation [44] and its involvement in epithelial responses to microbiota-associated metabolites [45], which are relevant to the barrier and microbial–metabolic outcomes assessed here. Western blot analysis further demonstrated that the protein levels of ASBT, PPARγ, and NLRP6 were significantly increased by PFE compared with the MOD group, although they remained lower than those in the CK group. ASBT is involved in intestinal bile acid transport [46]; however, the changes detected in colonic ASBT abundance do not by themselves establish altered functional bile acid uptake. Overall, comparison across the microbiota, metabolomic, and transcriptomic datasets revealed broadly parallel changes in microbial composition, cecal bile acid- and lipid-related metabolites, and host gene and protein expression. The exploratory genus-metabolite correlations did not remain significant after group adjustment and multiple-testing correction, limiting evidence for associations independent of treatment-group differences. These associations provide potential mechanistic clues but require further functional validation, particularly to determine whether NLRP6 contributes to the protective effects of PFE.

Several limitations of this study should be acknowledged. First, microbiota, metabolomic, transcriptomic, and protein analyses were performed only in the high-dose PFE group, limiting interpretation of the dose dependence of the associated molecular changes. Second, RNA-seq and Western blot analyses included three independent biological replicates per group, which may limit sensitivity to smaller expression changes and the precision of estimates. The inclusion of only male mice may also limit the generalisability of the findings. In addition, the metabolites were putatively annotated using non-targeted metabolomics, and targeted quantification of the identified bile acids was not performed. The associations observed across the microbiota, metabolite, and host datasets therefore do not establish causal relationships. The effects of heating during extraction on individual phytochemicals were not directly assessed; therefore, possible heating-related changes in extract composition cannot be excluded. The pretreatment design primarily supports investigation of PFE as a potential preventive dietary intervention, rather than establishing efficacy against existing colitis. Future studies initiating PFE administration only after colitis onset are needed to assess its therapeutic potential. Further studies using larger independent cohorts, targeted bile acid analysis, microbiota manipulation, and pathway-specific interventions are required to clarify the underlying mechanisms and identify the phenolic constituents principally responsible for the effects of PFE.

**Figure 11:**
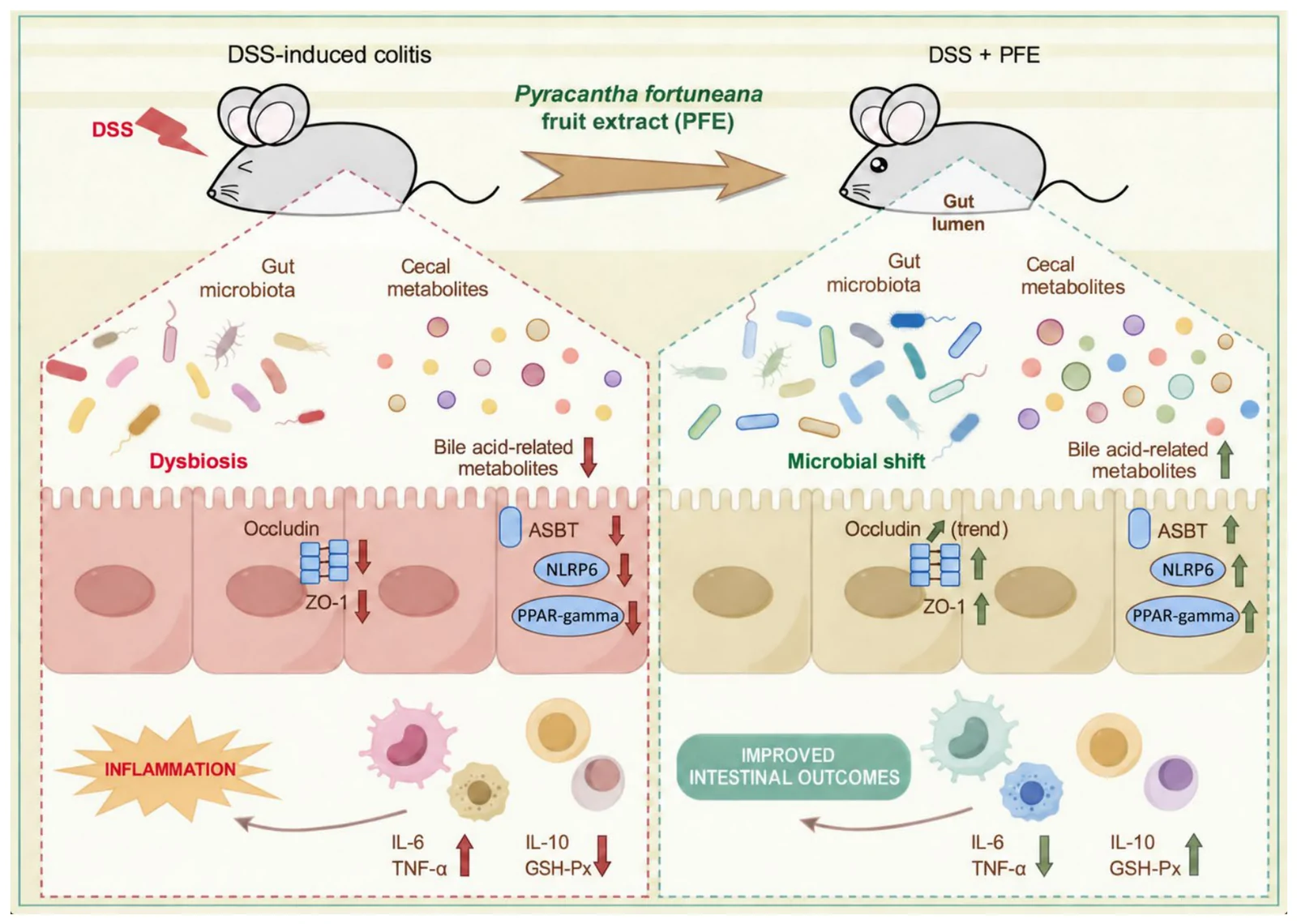
Proposed overview of PFE-associated changes in DSS-induced colitis, involving the gut microbiota, cecal metabolites, bile secretion- and PPAR-related responses, inflammatory cytokines, and intestinal barrier markers. The bacterial and metabolite symbols are schematic representations of the gut microbial community and cecal metabolite pool, respectively, and do not represent individual bacterial taxa or specific metabolites. Arrows indicate the observed directions of change and do not imply causality.

Based on the present findings, Figure 11 provides a proposed overview of the biological changes associated with PFE treatment in DSS-induced colitis. PFE alleviated several clinical and pathological manifestations of colitis and modulated inflammatory cytokines and serum GSH-Px levels. These effects were accompanied by increased microbial diversity and evenness and a distinct shift in gut microbial composition relative to the MOD group. PFE treatment was also associated with higher relative levels of selected bile acid-related metabolites and changes in lipid-related metabolites, together with transcriptional alterations in bile secretion- and PPAR-related pathways. At the protein level, PFE partially increased ASBT, PPARγ, and NLRP6 expression. Moreover, PFE restored ZO-1 expression, tended to increase Occludin expression, and increased colonic DAO levels, indicating partial improvement in intestinal barrier-related parameters. It should also be recognized that PFE is a complex plant extract, and uncharacterized components, including residual carbohydrates and other non-phenolic constituents, may also contribute to the observed microbial changes; therefore, these effects cannot be attributed exclusively to the identified phenolic compounds. In addition, the intestinal fate of PFE-derived phytochemicals was not specifically investigated, and the present non-targeted metabolomic analysis cannot distinguish parent PFE constituents from their intestinal or microbial metabolites. Further compositional, fractionation, and targeted intestinal or fecal analyses are needed to clarify the contributions and biotransformation of individual PFE constituents. Collectively, these findings suggest that the protective effects of PFE are associated with changes in gut microbial composition, cecal metabolism, and host molecular responses. The phenolic-rich nature of PFE further supports its potential for investigation as a food-derived bioactive ingredient, although its active constituents and causal mechanisms require additional validation.

## 5. Conclusions

In conclusion, phenolic-rich PFE attenuated DSS-induced colitis in mice, as evidenced by reduced disease activity, improved colonic morphology, modulation of inflammatory cytokines, and partial improvement in intestinal barrier-related parameters. PFE treatment was further associated with changes in gut microbial diversity and composition, cecal bile acid- and lipid-related metabolite profiles, and colonic molecular responses involving bile secretion- and PPAR-related pathways. Collectively, these findings suggest that microbial, metabolic, and host responses may be involved in the protective effects of PFE, although the present data do not establish causal relationships among these changes. Further targeted metabolite quantification and functional intervention studies are needed to identify the principal active phenolic constituents and validate the proposed mechanisms. These findings provide experimental support for further investigation of PFE as a potential plant-derived bioactive ingredient for intestinal health.

## Figures and Tables

**Table 1:** Representative phenolic compounds identified and quantified in PFE by HPLC–UV.

No.	Compounds	Molecular Formula	Class	RT (min)	Contents (mg/g DW)
1	Procyanidin B1	C_30_H_26_O_12_	Proanthocyanidins (condensed tannins)	18.65	0.825 ± 0.185
2	Cryptochlorogenic acid	C_16_H_18_O_9_	Phenolic acids	21.56	0.171 ± 0.037
3	Chlorogenic acid	C_16_H_18_O_9_	Phenolic acids	22.67	0.453 ± 0.306
4	Procyanidin B4	C_30_H_26_O_12_	Proanthocyanidins (condensed tannins)	25.23	1.048 ± 0.542
5	Procyanidin B2	C_30_H_26_O_12_	Proanthocyanidins (condensed tannins)	25.69	1.261 ± 0.112
6	Epicatechin	C_15_H_14_O_6_	Flavonoids	26.74	0.580 ± 0.049
7	Quercetin-3-O-rutinoside (Rutin)	C_27_H_30_O_16_	Flavonoids	51.10	5.118 ± 0.342
8	Quercetin-3-O-galactoside (Hyperoside)	C_21_H_20_O_12_	Flavonoids	51.84	0.818 ± 0.243
9	Quercetin-3-O-glucoside (Isoquercitrin)	C_21_H_20_O_12_	Flavonoids	52.25	2.401 ± 0.886

## Data Availability

The original contributions presented in this study are included in the article/Supplementary Material. Further inquiries can be directed to the corresponding authors.

## Supplementary Materials

The following supporting information can be downloaded at: https://www.mdpi.com/article/10.3390/foods15193528/s1, Figure S1: Chemical characterization of PFE by HPLC–UV and UPLC–ESI–QTRAP–MS/MS; Figure S2: Body weight changes during DSS-induced colitis and PFE intervention; Figure S3: Differential metabolite analysis; Figure S4: Exploratory correlations between bacterial genera and putatively annotated bile acid-related metabolites across 18 matched mice from the CK, MOD, and PFE groups (*n* = 6 per group); Table S1: Disease activity index (DAI) scoring system; Table S2: Gradient elution conditions for liquid chromatography; Table S3: Mass spectrometry parameters for non-targeted metabolomic analysis.

## Abbreviations

The following abbreviations are used in this manuscript:

UC	Ulcerative colitis
PFE	*P. fortuneana* fruit extract
DSS	Dextran sodium sulfate
IBD	Inflammatory bowel disease
TNF	Tumor necrosis factor
IL	Interleukins
ZOs	Zonula occludens
5-ASA	5-Aminosalicylic acid
IL-10	Interleukin-10
DAO	Diamine oxidase
GSH-Px	Glutathione peroxidase
IL-6	Interleukin-6
H&E	Hematoxylin and eosin
HPLC	High-performance liquid chromatography
DAD	Diode array detector
IACUC	Institutional Animal Care and Use Committee
CK	Control group
MOD	Model group
LPFE	Low-dose PFE group
MPFE	Medium-dose PFE group
HPFE	High-dose PFE group
CMC-Na	Carboxymethyl cellulose sodium
ASVs	Amplicon sequence variants
ANOVA	Analysis of variance
TPC	Total phenolic content
