# Supplementary material for: Phenolic-Rich Extract from Pyracantha fortuneana Fruit Alleviates DSS-Induced Colitis in Mice: Insights from Gut Microbiota, Metabolomic, and Transcriptomic Analyses
Source: Foods. 2026 Oct 2;15(19):3528. doi: 10.3390/foods15193528 (PMC13665218; doi:10.3390/foods15193528)
Supplement: Supplementary file 1 [file foods-15-03528-s001.zip › foods-4580243-supplementary.pdf]

Supplementary Material

Figure S1. Chemical characterization of PFE by HPLC–UV and UPLC–ESI–QTRAP–MS/MS. (A) Representative HPLC–UV chromatogram of PFE recorded at 280 nm. Peaks 1–9 correspond to procyanidin B1 (1), cryptochlorogenic acid (2), chlorogenic acid (3), procyanidin B4 (4), procyanidin B2 (5), epicatechin (6), rutin (7), hyperoside (8), and isoquercitrin (9), respectively. (B) Representative total ion chromatograms obtained by UPLC–ESI–QTRAP–MS/MS in positive- and negative-ion modes.

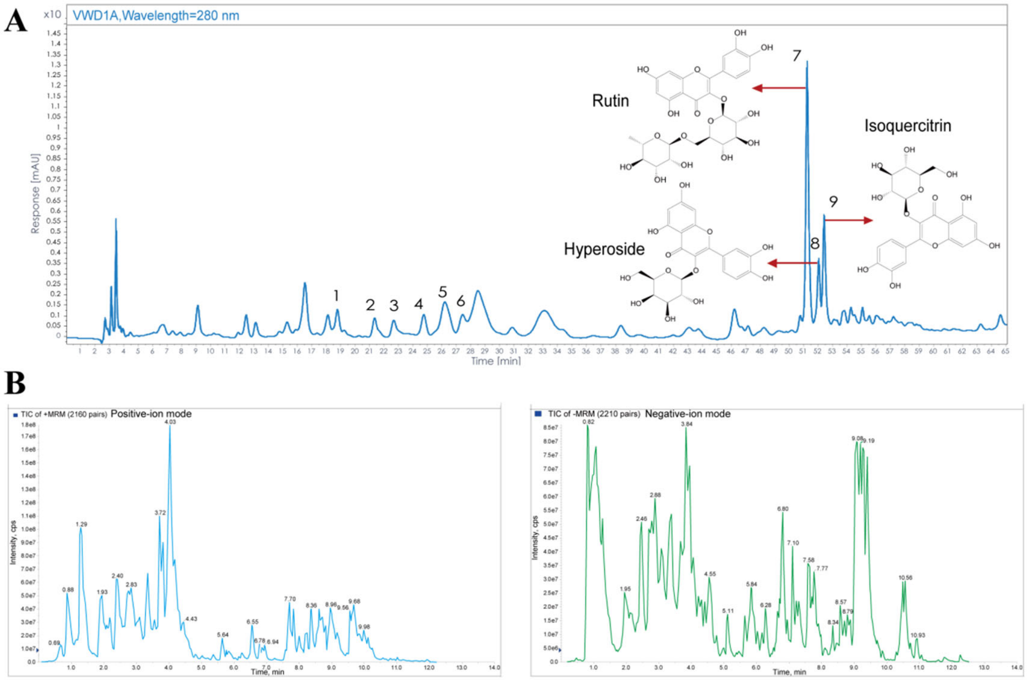

Figure S2. Body weight changes during DSS-induced colitis and PFE intervention. No significant differences were observed among groups.

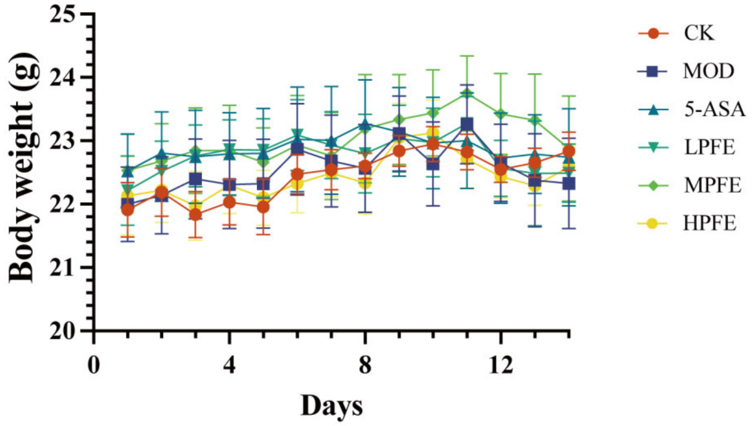

Figure S3. Differential metabolite analysis. (A) Venn diagram of differential metabolites between the MOD vs. CK and PFE vs. MOD groups. (B) Differential metabolites associated with lipid metabolism identified based on KEGG annotation. (C) KEGG enrichment of co-regulated metabolites in the CK vs. MOD and MOD vs. PFE groups.

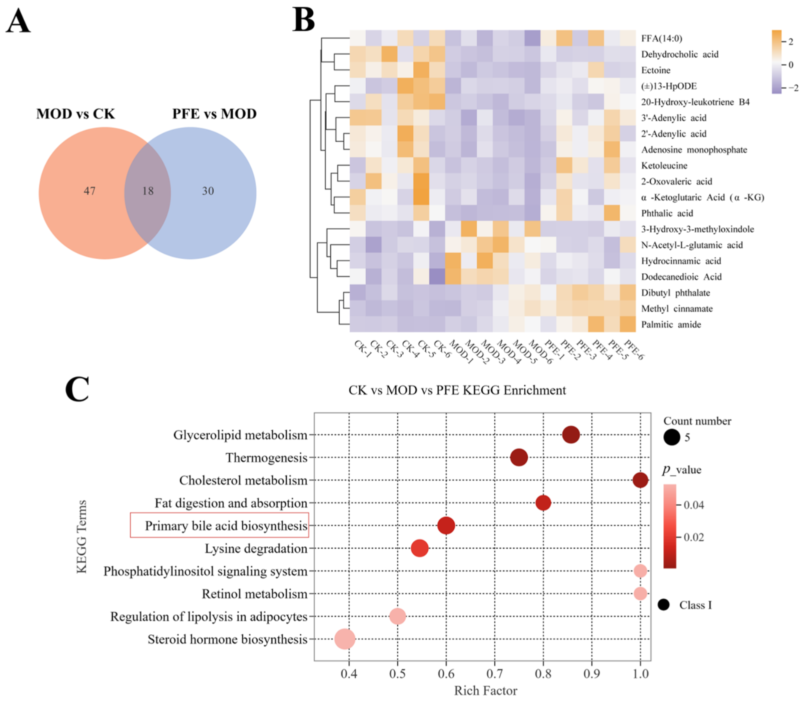

Figure S4. Exploratory correlations between bacterial genera and putatively annotated bile acid-related metabolites across 18 matched mice from the CK, MOD, and PFE groups (n = 6 per group). Colours represent Spearman correlation coefficients. *q < 0.05 after Benjamini–Hochberg correction of two-sided permutation P values across 260 comparisons. PFE denotes the high-dose treatment group.

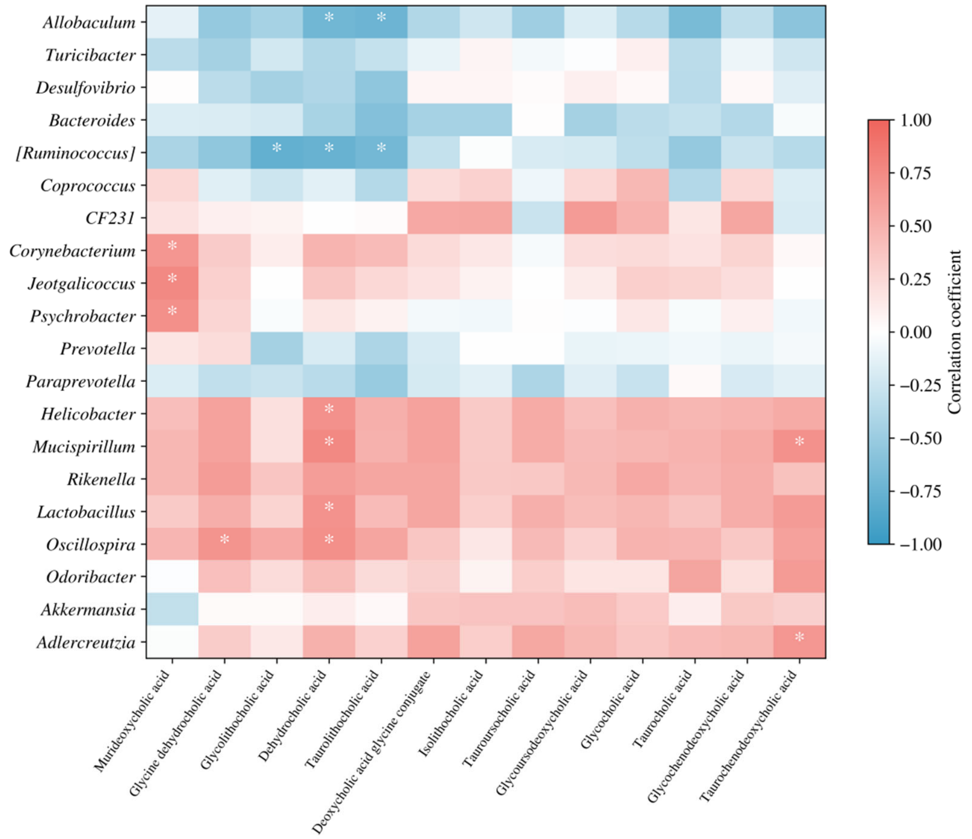

Table S1. Disease activity index (DAI) scoring system

Score	Weight loss (%)	Stool consistency	Blood in feces
0	None	Normal	Negative (no bleeding)
1	1-5%	Loose stools	Negative
2	5-10%		Hemoccult positive (slight)
3	10-20%	Diarrhea	Hemoccult positive
4	>20%		Gross bleeding

Table S2. Gradient elution conditions for liquid chromatography.

Time (min)	Phase A (%)	Phase B (%)
0	95	5
2	80	20
5	40	60
6	1	99
7.5	1	99
7.6	95	5
10	95	5

Table S3. Mass spectrometry parameters for non-targeted metabolomic analysis.

Parameter	ESI+	ESI-
Acquisition time (min)	10	10
Ionization voltage (V)	5000	-4000
Ion source temperature (° C)	550	450
Nebulizer gas (psi)	50	50
Heater gas (psi)	60	60
Curtain gas (psi)	35	35
Declustering potential (V)	60	-60
MS1 collision energy (V)	10	-10
MS2 collision energy (V)	30	-30
Collision energy step size (V)	15	15
MS1 mass range (Da)	50 – 1000	50 – 1000
MS2 mass range (Da)	25 – 1000	25 – 1000
MS1 acquisition time (s)	0.2	0.2
MS2 acquisition time (s)	0.04	0.04
Number of candidate ions	18	18
Ion exclusion logic	Always, For 3 seconds, After 3 occurrences	Always, For 3 seconds, After 3 occurrences
